# Strengthening the Partnerships That Promote Health Equity and Social Justice

**DOI:** 10.1089/hs.2023.0012

**Published:** 2023-09-27

**Authors:** John Auerbach, Alice T. Chen

**Affiliations:** John Auerbach, MBA, is Senior Vice President, Public Health, ICF, Reston, VA.; Alice T. Chen, MD, is Former Senior Advisor, Made to Save, Civic Nation, Washington, DC.

**Keywords:** COVID-19, Public health preparedness/response, Health equity, Citizen engagement, Epidemic management/response

## Background

The public health sector in the United States is in a moment of reimagining how it protects the health of the public during an emergency as well as in more routine times. While increased funding for public health is crucial, the sector also faces some key challenges that require a significant reorientation toward a more community-centered approach. Our communities are increasingly diverse, and many communities live with complex, ongoing, and historical inequities that negatively impact their ability to respond to crises and their openness to trusting government efforts. Misinformation and disinformation are increasingly easy to disseminate.

To rise to the challenge of addressing health security in the context of this shifting landscape, the public health sector must develop genuine and sustained partnerships with local organizations and residents—especially in chronically underserved communities—to create a network that can address longstanding health inequities while serving as a rapid response force in times of crisis. As a result of the COVID-19 pandemic, efforts toward laying the groundwork for greater partnership have already begun. Many community organizations took on a new or expanded role in addressing health, starting with COVID-19 concerns but often expanding into general health and social determinants of health. At the same time, as health practitioners watched the disproportionate devastation in areas with populations at higher risk, they gained a greater appreciation of the need to elevate equity as a central public health concern.

Despite these gains, the public health sector largely lacks well-established partnerships in communities at high risk. Relatively few governmental resources reach small neighborhood-specific organizations or trusted local leaders. Neither the US Centers for Disease Control and Prevention (CDC) nor state and local public health associations keep track of the total amount of funding that reaches community-based organizations; however, few governmental public health programs specifically target grants to community-based groups. To be fair, some of the more significant obstacles to funding community organizations are beyond the control of public health agencies.

Yet this is an ideal moment to overcome those obstacles. The public health sector together with policymakers can build on the experiences from the pandemic to reshape *how* public health approaches community partnerships to promote health equity and security.

## Who Are the Populations at Elevated Risk?

The populations who have been historically marginalized and who remain at elevated risk for preventable illness, injuries, and deaths include Black, African American, American Indian/Alaskan Native, Latino, Asian and other populations of color as well as immigrant populations, people with low incomes, people with disabilities and older adults with underlying health conditions, LGBTQ (lesbian, gay, bisexual, transgender, queer) populations, and those living in rural and/or frontier settings. Virtually all of these groups experienced excess negative impacts from COVID-19, often including higher rates of hospitalization and death compared with the general population.^1-4^ Similar disparities exist for many other health indicators such as HIV, heart disease, cancer, asthma, suicide, violence, and behavioral health.^5^

The elevated risk is largely a result of social, economic, and environmental factors rather than individual behavior. For example, a disproportionate number of people of color and low-income people held jobs that increased their likelihood of COVID-19 infection—such as those in food processing and packing facilities and grocery and other retail stores.

## Barriers to Partnerships Between Public Health and Community Organizations

Despite a growing recognition of the value of community partnerships, there has been relatively limited progress in nurturing them. Barriers to effective partnerships include lack of trust and lack of funding and resources for community partners.

Feedback from community organizations during the pandemic revealed common themes of mistrust of government and healthcare due to historic and present-day inequities and structural racism.^6^ Groups on the frontlines in local communities lacked avenues to share their expertise with public health departments. Often public health officials disseminated information and plans without inviting these groups to participate in collaborative planning and problem solving.

Lack of funding, direct or indirect, remains the most noteworthy obstacle to the meaningful involvement of community organizations in public health activities. The general rule among policymakers is that public health funding is intended for governmental health departments or their associations, without additional intentional investments in community organizations. Congress often includes language within line items in CDC and other federal agency budgets that determines who receives funding, with state, tribal, territorial, and large local health departments as the frequent recipients. The language may even include a preset formula to guide funding allocation.^7^ Such formulas and priorities may exist in order to provide coverage to all municipalities, and to recognize the specific roles of each agency. However, a frequent result of these restrictions and the lack of complementary funding for community organizations is the failure to reach people in communities at high risk as noted earlier.

Community organizations face other obstacles to public funding. For example, they may not have administrative or budgetary prerequisites to fulfill complex application and reporting requirements, or they may lack a track record of prior public funding. Those who serve communities that speak few if any common languages may find that the application process places them at a competitive disadvantage. Others require more technical assistance than is readily available to help them adapt their capacities to funded efforts.

In addition, funders may simply find it faster and easier to rely upon large organizations with established grants and track records—especially when under pressure to allocate and expend funds as quickly as possible, even when it would be preferable to allow community-based organizations to apply. This was notably the case in the response to the COVID-19 pandemic when there was great pressure to distribute funds rapidly. For example, CDC's $2.25 billion National Initiative to Address COVID-19 Health Disparities Among Populations at High-Risk and Underserved, Including Racial and Ethnic Minority Populations and Rural Communities was a groundbreaking effort to address the disproportionate burden of disease in marginalized communities. It funded only state, territorial, and large local public health departments in part because this was a quick and well-trod approach to distributing funding quickly. Some of the resources may have reached local community-based organizations, but because grantees are not required to fund community-based subrecipients or to highlight whether they did, there is no publicly available data to assess how much of the $2.25 billion reached community-based organizations.^8^

Another obstacle is short-term or disease- and condition-specific funding that makes blending and braiding—layering multiple funding streams to support a single initiative—difficult, if not impossible. These restrictions result in the administrative and operational burdens of managing differing timelines, grant rules, reporting requirements, and hiring requirements and availability. Restricted grant opportunities also dictate what the community should focus on, thus limiting the community organizations' abilities to set their own priorities. In a powerful example of overcoming these challenges, the Rhode Island Health Department creatively linked funds from several state and federal grants and programs to create Health Equity Zones, which successfully supported community-led projects targeted at community-identified priorities.^9^ However, the process was difficult to replicate because it required significant advance planning and extensive negotiations with the CDC and other funders.^10^

## Positive Examples in Government

There are models of federal public health funding reaching communities and community-based organizations that could be expanded. For example, the CDC's Racial and Ethnic Approaches to Public Health (REACH) program works to “reduce health disparities among specific racial and ethnic groups in communities with the highest risk or rates of chronic disease.”^11^ The program encourages applications from organizations with strong community ties. Awardees often hire people from the communities served who adapt activities and materials to specific cultures, languages, and practices.

Another CDC initiative is more explicit about getting funds to community organizations. The Comprehensive High-Impact HIV Prevention Programs for Young Men of Color Who Have Sex With Men and Young Transgender Persons of Color program funds are targeted “for community-based organizations (CBOs) to develop and implement high-impact human immunodeficiency virus (HIV) prevention programs.”^12^ The HIV programs further require applicants to establish a new or enhance an existing Community Engagement Group (CEG) [or Consumer Advisory Board (CAB) to assist with program planning, implementation, and evaluation efforts.

This language and these ideas could easily apply to other initiatives. The CDC also recently offered another adaptable approach. In its notice of funding opportunity for the Strengthening US Public Health Infrastructure, Workforce, and Data Systems, state health agencies were required to provide 40% of their funded awards to local jurisdictions not otherwise funded.^13^ Future CDC grant opportunities might apply such percentage subcontracting requirements to CBOs. In addition, grant requirements could mandate that applicants provide administrative support and other services to subcontractors as needed.

## Insights From Efforts to Support Community Organizations Outside of Government

Outside the constraints of government, Made to Save, a national initiative of the nonprofit group Civic Nation, led an 18-month national campaign that supported CBOs and trusted messengers in conducting COVID-19 vaccine outreach and education. With $15 million in private funding, it built a support infrastructure and awarded $7 million in grants to 110 community-based groups, more than 80% of which were led by people of color.^14^

As stated in its final report, Made to Save “centered power within communities of color and within unvaccinated people themselves.”^14^ The initiative preferentially hired staff who had existing ties to the communities. They then identified high-need counties and CBOs that had community trust and track records of service, including many who were well established in their communities but were focusing on public health for the first time, spurred by the pandemic. The initiative provided technical assistance and support to potential grantees in designing their action plans and deliverables. When groups on the ground were unable to be primary grantees, Made to Save worked with state or regional coalitions to subcontract to them.

Once funded, Made to Save grantees received training and support based on their needs including public health updates, data, communications and media outreach, and digital organizing. Made to Save also formed a multisector coalition that met regularly to offer information, resources, a forum to raise issues and share successes, and access to federal policymakers—who themselves benefited from having a vehicle for hearing from those in the field.^14^

This multifaceted support enabled grantees to adapt public health messages and strategies to local environments to overcome deep-seated mistrust and barriers to access.^14^ The International Mayan League hired Mayan health promoters who were able to connect with Indigenous Mayan immigrants in the Washington, DC, area in their native Ixil. Proyecto Vida Digna canvassed 10 low-income Latino unincorporated communities, or *colonias*, in Texas, speaking with tens of thousands of residents and partnering with a Federally Qualified Health Center to schedule vaccine appointments.

Lessons from Made to Save, such as building flexibility into contracts, can be adapted for federal, state, and local public health entities. This flexibility enabled grantees to adapt to changing circumstances while meeting the goals of the grant. Similarly, flexibility in measuring impact allowed grantees to conduct the work that was best suited for their communities.

At the state and local levels, some public health departments developed similar innovative partnerships with CBOs to address challenges during the COVID-19 pandemic and could serve as examples for other public health issues and other departments. For example, Denver-area public health departments partnered with the nonprofit Colorado Health Institute to create a microgrant program that set topline public health messaging goals but allowed trusted sources within communities to determine how best to deliver those messages.^15^ They have now adapted the program to address stigma around mental health. Similarly, the Chicagoland Vaccine Partnership worked with nonprofit Partners in Health, City College of Chicago, and the Chicago Department of Public Health to train people to be trusted COVID-19 vaccine ambassadors in their own communities.^16^ They also provided vaccine education grants to 60 community organizations that have deep trust in communities but are not usually associated with public health, such as churches, youth boxing programs, and violence prevention organizations.

## Recommendations Going Forward

Lessons from the COVID-19 pandemic and examples of efforts from Made to Save and several public health departments reinforce the importance and feasibility of strong community partnerships to meet the needs of historically marginalized groups. Through these partnerships, public health and CBOs can work together to strengthen community resources and resilience, which are central to our ability to respond to the next pandemic, bioterrorism event, weather event, or ongoing health challenges. These efforts are also crucial for moving forward on addressing longstanding disparities in health and beyond. To make progress, we offer the following recommendations:

- Public health agencies and policymakers should prioritize funding grassroots CBOs within marginalized neighborhoods.
- Funders should simplify the application process to increase the likelihood that community organizations with limited infrastructure capacity or less familiarity with the process will be able to apply. At all times, but especially when complexity cannot be reduced, training and technical assistance to the potential applicants should be offered.
- Funding for community organizations should be sustained over time, not just during emergencies.
- Whenever possible, funding for community organizations should be allowed to address a wide range of health issues rather than only a single issue. Ideally, community members themselves should help determine the issues to address as well as how to address them.
- Where direct funding of community organizations is not feasible, steps should be taken to fund them as subcontractors. Among the approaches to consider are fixed percentages of funding for CBOs. In addition, the directly funded organizations that subcontract with them should receive aid to allow them to offer mentoring and other support.
- Steps should be taken to ensure that CBOs and individuals from marginalized communities have ongoing mechanisms for meaningful input into the development of policies and programs that have a disproportionate impact on their populations and that they are treated as true partners and leaders in these efforts.

## Conclusion

To fulfill its mission to safeguard the health of the population, the public health sector needs to form strong partnerships with people and groups within communities at higher risk. If the health sectors and policymakers take action to develop and sustain these partnerships, the nation will be better equipped to avert illness and death among our more vulnerable populations.
